# Neurological Manifestations and High Viral Load as Independent Predictors of Mortality in Severe Fever With Thrombocytopenia Syndrome

**DOI:** 10.1093/ofid/ofaf803

**Published:** 2025-12-30

**Authors:** Rujia Chen, Yutong Xing, Wei Wei, Yun Wang, Ting Wang, Renren Ouyang, Shiji Wu, Feng Wang, Hongyan Hou

**Affiliations:** Department of Laboratory Medicine, Tongji Hospital, Tongji Medical College, Huazhong University of Science and Technology, Wuhan Hubei Province, China; Department of Laboratory Medicine, Tongji Hospital, Tongji Medical College, Huazhong University of Science and Technology, Wuhan Hubei Province, China; Department of Laboratory Medicine, Tongji Hospital, Tongji Medical College, Huazhong University of Science and Technology, Wuhan Hubei Province, China; Department of Laboratory Medicine, Tongji Hospital, Tongji Medical College, Huazhong University of Science and Technology, Wuhan Hubei Province, China; Department of Laboratory Medicine, Tongji Hospital, Tongji Medical College, Huazhong University of Science and Technology, Wuhan Hubei Province, China; Department of Laboratory Medicine, Tongji Hospital, Tongji Medical College, Huazhong University of Science and Technology, Wuhan Hubei Province, China; Department of Laboratory Medicine, Tongji Hospital, Tongji Medical College, Huazhong University of Science and Technology, Wuhan Hubei Province, China; Department of Laboratory Medicine, Tongji Hospital, Tongji Medical College, Huazhong University of Science and Technology, Wuhan Hubei Province, China; Department of Laboratory Medicine, Tongji Hospital, Tongji Medical College, Huazhong University of Science and Technology, Wuhan Hubei Province, China

**Keywords:** neurological symptoms, prognosis, severe fever with thrombocytopenia syndrome, severe fever with thrombocytopenia syndrome virus

## Abstract

**Background:**

Severe fever with thrombocytopenia syndrome (SFTS), an emerging tick-borne viral hemorrhagic fever, is characterized by high mortality rates. While neurological complications (eg, seizures, encephalitis) have been identified as adverse prognostic factors in severe cases, their association with viral replication, immune responses, and neuroinflammation remain poorly defined and urgently require systematic investigation.

**Method:**

A cohort of 277 patients with SFTS was included and stratified based on neurological symptoms. Clinical characteristics, laboratory results, and immune markers were compared between groups.

**Results:**

Neurological symptoms developed in 78 (28.2%) patients and were associated with significantly higher 28-day mortality. These patients had higher viral loads, elevated inflammatory cytokines (IL-6, IL-10, TNF-α, and ferritin), and more severe multi-organ dysfunction. Compared with survivors, nonsurvivors showed reduced platelet and T-cell counts, and disregulated B-cell subsets with increased plasmablasts and double-negative B cells. Viral load correlated with cytokine elevation, coagulopathy (prolonged APTT), and renal impairment (reduced eGFR). Multivariate Cox proportional hazards regression identified neurological symptoms (HR = 2.565; 95% CI: 1.641–4.011; *P* < .001) and viral load (HR = 1.785 per log₁₀ increase; 95% CI: 1.503–2.120; *P* < .001) as independent predictors of mortality.

**Conclusions:**

Neurological manifestations and elevated viral load play a central role in the progression of SFTS and are closely associated with adverse clinical outcomes. Considering neurological symptoms and immune profiles in prognostic assessments may improve early recognition of high-risk patients and inform clinical management.

Severe fever with thrombocytopenia syndrome (SFTS), caused by the SFTS virus (SFTSV), is an emerging tick-borne infectious disease first identified in rural China in 2009 [1]. Since then, SFTS has been reported in several countries across Asian, including South Korea, Japan, and Vietnam [2–4]. The disease typically presents with fever, thrombocytopenia, leukopenia, gastrointestinal symptoms, and, in severe cases, multiple organ dysfunction syndrome (MODS) [5]. From 2009 to 2019, SFTSV outbreaks resulted in more than 13 000 cases in mainland China and over 1000 cases in South Korea, with the mortality rate ranging from 12% to 30% [6–8]. Although the epidemiology and clinical manifestations of SFTS are increasingly documented, the mechanisms underlying its variable severity, particularly the role of central nervous system (CNS) involvement, remain insufficiently understood.

Patients with SFTS often exhibit dysregulated immune response and excessive inflammatory response [9]. This cytokine storm contributes to systemic inflammatory response syndrome, leading to vascular leakage, tissue damage, multiple organ dysfunction, and increased mortality rate [10]. Laboratory findings in patients with SFTS commonly include thrombocytopenia, leukopenia, elevated liver enzymes, and coagulation abnormalities [11, 12]. These parameters reflect both viral replication and immune-mediated damage. Hemophagocytic lymphohistiocytosis (HLH) is a life-threatening condition characterized by excessive activation of the immune system, leading to hemophagocytosis in bone marrow and other tissues [13]. This phenomenon contributes to pancytopenia and organ failure. Early recognition and management of HLH are vital to improving survival rates in patients with SFTS [14]. High viral loads, especially during the early stages of infection, are associated with severe disease manifestations [15]. Quantitative assessments of viral load can serve as prognostic indicators, aiding in early identification of patients at risk for adverse outcomes.

Neurological manifestations, such as seizures, cognitive impairment, and consciousness disorders, are increasingly recognized as critical factors influencing the prognosis of SFTS. Patients who develop encephalitis as a complications of SFTS face a mortality rate exceeding 50% [16]. Research has identified several risk factors for CNS complications, including elevated SFTSV RNA levels and lactate dehydrogenase (LDH) levels during the fever stage, both of which have been independently associated with increased risk of severe neurological outcomes [17, 18]. In addition, studies have shown elevated levels of pro-inflammatory cytokines, such as IL-6 and IL-8, in both serum and cerebrospinal fluid (CSF) of patients. This suggests that cytokine storms may contribute to increased blood-brain barrier permeability, thereby facilitating viral invasion of the CNS [19]. However, the associations between neurological manifestations and both viral load and laboratory findings still require further investigation. Elucidating the pathophysiology of CNS complications is crucial for developing targeted therapeutic interventions to improve clinical outcomes.

## METHODS

### Study Population

This retrospective cohort study included patients diagnosed with SFTS at Tongji Hospital, Wuhan, between March 2022 and August 2024. A total of 277 patients were included in the study. The inclusion criteria encompassed individuals who met the diagnostic criteria for SFTS, including acute fever, thrombocytopenia, and the detection of SFTSV RNA in plasma using polymerase chain reaction (PCR) [12]. Detailed demographic, clinical, and laboratory data were collected for all patients, and the study aimed to analyze various factors influencing the prognosis of SFTS. The patients with SFTS at the acute phase of disease were divided into acute survival (AS) group and acute deceased group (AD). The clinical outcomes of the patients were monitored for a period of 28 days from the onset of symptoms. Neurological symptoms in patients with SFTS were defined as the presence of acute altered mental status, seizures, cognitive impairment, or coma, without other identifiable causes such as metabolic, hepatic, or drug-induced encephalopathy. Classification was primarily based on clinician-documented observations within 24 hours of hospital admission. When available, CSF analysis and neuroimaging findings were used to support the diagnosis of CNS involvement (eg, encephalopathy or encephalitis). These criteria were utilized to stratify patients into neurological and non-neurological groups for subsequent comparative analyses. This study was approval by the ethical committee of Tongji Hospital, Tongji Medical College, Huazhong University of Science and Technology (TJ-IRB20230632). Written informed consent was obtained from all participants. The study protocol adheres to the ethical standards of the Declaration of Helsinki.

### Collection and Detection of Laboratory Indicators

Blood samples were collected upon hospitalization, complete blood count, liver and renal function tests, coagulation profiles, and cytokine levels were detected. The clinical data, including demographic information, underlying diseases, clinical symptoms and laboratory findings, comorbidity, treatment, and overall prognosis, were collected through the electronic medical record system. SFTSV RNA levels were quantified using the Da'an Gene SFTSV Fluorescent Quantitative PCR Detector Kit (Da An Gene Co., Ltd., Guangzhou, China), which targets the nucleoprotein gene of SFTSV. Quantitative PCR was performed strictly according to the manufacturer's instructions. The reported limit of detection for this assay is 100 copies/mL, as validated in the manufacturer's performance report and widely used in routine clinical diagnostics.

### Flow Cytometry Analysis

Fresh whole blood was collected within 24 hours of admission. The percentages and absolute numbers of T, B, and NK cells were determined using TruCOUNT tubes and BD Multitest 6-color TBNK ReagentKit (BD Biosciences). The antibody panels used and analysis flow were based on the methods described in our previously published research [20].

### Statistical Analysis

Categorical data were compared using the Chi-square test. For quantitative data, independent *t*-tests or Mann–Whitney *U* tests were used for normally or non-normally distributed data, respectively. Kaplan–Meier survival curves were generated, and differences between groups were assessed using the log-rank test. Correlation analysis was performed using the cor function in R, with Pearson correlation coefficients applied for normally distributed data and Spearman correlation coefficients for non-normally distributed data. Heatmaps were created using the “pheatmap” R package to visualize gene or biomarker expression patterns. The prognostic factors of 28-day overall survival were analyzed by Cox regression model. Variables with *P* < .05 in univariate analysis and/or strong clinical relevance were pre-specified for inclusion. Backward stepwise selection was performed based on the Akaike information criterion, with neurological manifestations and viral load forced into the model due to their clinical significance. Multicollinearity was assessed using variance inflation factors (VIF), and variables with VIF >5 were excluded or combined when appropriate. The proportional hazards assumption was tested using Schoenfeld residuals, and no significant violations were detected. Hazard ratios (HRs) and 95% confidence intervals (CIs) were reported. Statistical analyses were performed using GraphPad Prism version 9.5 (San Diego, CA), SPSS version 22.0 22.0 (IBM Corp., Armonk, NY). Statistical significance was determined as *P* < .05.

## RESULTS

### Baseline Characteristics of SFTS Patients With and Without Neurological Symptoms

A total of 277 patients diagnosed with SFTS were enrolled, including 146 females (52.7%) and 131 males (47.3%). Clinical outcomes were evaluated over a 28-day period from symptom onset, during which 181 patients (65.3%) survived and 96 (34.7%) died. Neurological symptoms developed in 78 patients (28.2%). Compared with those without neurological symptoms, affected patients were significantly (median age: 69 vs 64 years, *P* = .002), and had a markedly higher 28-day mortality rates (67.95% vs 21.61%, *P* < .001) (Table 1). They also required more intensive interventions, including invasive mechanical ventilation (28.21% vs 4.52%, *P* < .001) and continuous renal replacement therapy (32.05% vs 10.55%, *P* < .001). In addition, patients with neurological involvement had a higher prevalence of comorbidities, such as hypertension (42.31% vs 26.63%, *P* = .017) and hemophagocytosis (53.85% vs 31.16%, *P* = .001), indicating more severe immune dysregulation.

**Table 1.** Comparison of Baseline Characteristics in SFTS Patients With and Without Neurological Symptoms at Admission

Parameters	Total (n = 277)	No Neurological Symptoms (n = 199)	Neurological Symptoms (n = 78)	*P* Value
Age, median [Q1–Q3]	65.000 [57.000; 71.000]	64.000 [56.000; 70.000]	69.000 [61.000; 73.000]	.002
Sex, n (%)				1.000
Female	146 (52.708%)	105 (52.764%)	41 (52.564%)	
Male	131 (47.292%)	94 (47.236%)	37 (47.436%)	
Outcomes, n (%)				<.001
Survival	181 (65.343%)	156 (78.392%)	25 (32.051%)	
Deceased	96 (34.657%)	43 (21.608%)	53 (67.949%)	
Hemophagocytosis, n (%)				.001
No	173 (62.455%)	137 (68.844%)	36 (46.154%)	
Yes	104 (37.545%)	62 (31.156%)	42 (53.846%)	
Hypertension, n (%)				.017
No	191 (68.953%)	146 (73.367%)	45 (57.692%)	
Yes	86 (31.047%)	53 (26.633%)	33 (42.308%)	
Hormone usage, n (%)				.007
No	165 (59.567%)	129 (64.824%)	36 (46.154%)	
Yes	112 (40.433%)	70 (35.176%)	42 (53.846%)	
Continuous renal replacement therapy, n (%)				<.001
No	231 (83.394%)	178 (89.447%)	53 (67.949%)	
Yes	46 (16.606%)	21 (10.553%)	25 (32.051%)	
Invasive mechanical ventilation, n (%)				<.001
No	246 (88.809%)	190 (95.477%)	56 (71.795%)	
Yes	31 (11.191%)	9 (4.523%)	22 (28.205%)	
D from symptom onset to admission, median (Q1–Q3)	7.000 [5.000; 8.000]	7.000 [5.000; 8.000]	7.000 [5.000; 8.750]	.468
Hospitalization time, median (Q1–Q3)	7.000 [4.000; 11.000]	8.000 [5.000; 11.000]	5.000 [2.000; 10.000]	.002

### Laboratory Markers Associated With Neurological Involvement

Patients with neurological complications exhibited significant abnormalities across multiple laboratory parameters (Table 2), reflecting extensive organ dysfunction, coagulation disturbances, metabolic derangements, and systemic inflammation. Elevated levels of cardiac troponin I, myoglobin, and aspartate aminotransferase (AST) levels indicated marked cardiac, muscular, and hepatic injury. Coagulation profiles revealed prolonged activated partial thromboplastin time (APTT) and elevated D-dimer levels, suggestive of a hypercoagulable state. Metabolic dysfunction was evident from hypoalbuminemia and decreased estimated glomerular filtration rate (eGFR), indicating renal impairment. Furthermore, markedly elevated levels of inflammatory cytokines, including IL-6, IL-10, tumor necrosis factor-alpha (TNF-α), and ferritin, highlighted a pronounced cytokine response. Collectively, these findings laboratory findings suggest a complex, multifactorial pathophysiological mechanism underlying neurological complications in SFTS.

**Table 2.** Laboratory Markers in Patients With SFTS Stratified by Neurological Involvement

Parameters	Total (n = 277)	No Neurological Symptoms (n = 199)	Neurological Symptoms (*n* = 78)	*P* Value
**Blood routine indicators**				
WBC (×109/L)	3.5 [2.1; 6.0]	3.2 [1.9; 5.5]	3.7 [2.5; 6.9]	**.046**
Lymphocyte counts (×109/L)	0.7 [0.4; 1.0]	0.6 [0.3; 1.0]	0.8 [0.5; 1.0]	**.028**
Lymphocytes %	19.5 [12.1; 34.5]	18.4 [11.9; 34.5]	20.6 [12.3; 34.7]	.498
Neutrophils (×109/L)	2.4 [1.2; 4.2]	2.1 [1.1; 3.9]	2.8 [1.4; 5.0]	.052
Neutrophils %	73.4 [56.0; 84.3]	73.9 [54.5; 84.3]	71.0 [59.4; 83.3]	.904
Platelet (×1012/L)	50.0 [32.0; 68.0]	50.5 [34.8; 69.0]	42.5 [28.3; 65.8]	.061
RBC (×1012/L)	4.3 (0.7)	4.3 (0.6)	4.2 (0.8)	.429
Hemoglobin (g/L)	129.4 (21.2)	130.0 (18.9)	127.8 (26.3)	.513
**Blood biochemistry indicators**				
ALT (U/L)	86.0 [46.0; 167.0]	81.5 [42.0; 159.3]	99.0 [57.5; 182.5]	.099
AST (U/L)	209.0 [100.0; 476.0]	187.0 [88.8; 419.0]	279.0 [167.0; 633.5]	**.001**
LDH (U/L)	740.0 [442.0; 1278.0]	641.5 [374.8; 989.3]	1264.0 [669.5; 1856.5]	**<.001**
Globulin (g/L)	27.9 [25.5; 30.4]	27.6 [25.4; 30.2]	28.4 [25.9; 30.6]	.289
Albumin (g/L)	33.3 (4.6)	34.3 (4.2)	30.8 (4.8)	**<.001**
TBIL (μmol/L)	9.0 [6.3; 12.8]	8.6 [6.2; 11.8]	10.0 [6.7; 16.1]	**.017**
IBIL (μmol/L)	3.9 [2.3; 5.9]	4.0 [2.5; 6.0]	3.8 [1.9; 5.2]	.14
DBIL (μmol/L)	4.4 [3.0; 6.9]	4.2 [2.9; 5.9]	5.1 [3.7; 10.9]	**.001**
HCO3− (mmol/L)	19.8 [17.4; 21.8]	20.3 [17.9; 22.0]	18.1 [15.6; 20.5]	**.001**
eGFR (ml/min/1.73 m^2^)	73.9 [51.4; 90.5]	79.2 [60.0; 92.6]	56.2 [33.2; 78.7]	**.001**
Creatinine (μmol/L)	83.0 [66.0; 116.0]	78.0 [63.5; 100.5]	106.0 [74.5; 156.0]	**.001**
Uric acid (μmol/L)	262.0 [200.8; 351.0]	251.0 [193.7; 329.0]	303.5 [221.8; 439.0]	**.002**
Urea (mmol/L)	6.1 [4.4; 9.1]	5.5 [4.0; 7.5]	9.0 [5.9; 14.5]	**.001**
Total cholesterol (mmol/L)	3.1 [2.5; 3.6]	3.1 [2.5; 3.6]	3.0 [2.5; 3.7]	.993
Triglyceride (mmol/L)	2.0 [1.5; 2.8]	2.0 [1.4; 2.6]	2.3 [1.6; 3.6]	**.024**
Lactic acid (mmol/L)	1.9 [1.3; 2.7]	1.6 [1.1; 2.6]	2.2 [1.7; 2.8]	**.003**
Myoglobin (ng/mL)	226.3 [122.1; 510.1]	195.1 [93.1; 370.3]	364.3 [189.4; 962.5]	**<.001**
cTnI (pg/mL)	107.3 [35.4; 314.4]	58.8 [27.1; 165.2]	244.0 [128.4; 851.5]	**<.001**
CK-MB (ng/mL)	3.0 [1.4; 6.9]	2.3 [1.2; 5.2]	5.6 [2.8; 9.3]	**<.001**
CK (U/L)	521.0 [199.0; 1288.0]	420.0 [178.5; 1269.3]	858.0 [401.0; 1415.0]	**.012**
Lipase (IU/L)	187.6 [97.0; 339.8]	162.6 [95.6; 280.4]	290.3 [133.7; 641.5]	**<.001**
Amylopsin (U/L)	83.0 [53.0139.0]	74.5 [50.0; 111.5]	121.5 [84.5; 244.5]	**<.001**
**Inflammatory indicators**				
hsCRP (mg/L)	4.6 [1.8; 12.9]	3.2 [1.5; 8.7]	10.1 [3.0; 26.9]	**<.001**
Ferritin (μg/L)	9832.0 [3250.7; 23 291.3]	6187.9 [2378.7; 15 991.0]	20 048.0 [10 198.0; 45 839.0]	**<.001**
IL-1β (pg/mL)	5.0 [5.0; 10.5]	5.0 [5.0; 7.8]	6.9 [5.0; 14.6]	**.003**
IL-2R (U/mL)	1217.5 [887.0; 1740.8]	1045.5 [779.7; 1486.3]	1684.0 [1127.0; 2335.0]	**<.001**
IL-8 (pg/mL)	25.1 [14.275; 61.250]	20.2 [13.1; 36.7]	59.2 [23.2; 203.8]	**<.001**
IL-10 (pg/mL)	47.7 [15.6; 151.0]	27.4 [12.9; 86.0]	139.5 [33.5; 227.0]	**<.001**
TGF-β1 (pg/mL)	4.1 [2.6; 7.3]	4.7 [2.7; 9.3]	4.7 [3.4; 9.2]	.820
TNF-α (pg/mL)	24.2 [16.2; 45.1]	21.3 [14.8; 32.9]	42.7 [21.9; 82.7]	**<.001**
IL-6 (pg/mL)	37.8 [12.7; 112.5]	29.3 [10.6; 63.8]	74.1 [33.7; 227.7]	**<.001**
PCT (ng/mL)	0.3 [0.1; 0.7]	0.2 [0.1; 0.5]	0.7 [0.2; 1.9]	**<.001**
**Coagulation markers**				
APTT (S)	54.2 [43.7; 65.2]	52.2 [42.2; 62.8]	56.6 [47.8; 76.8]	**.014**
PT (S)	12.8 [12.2; 13.5]	12.800 [12.1; 13.5]	13.0 [12.4; 13.9]	.051
TT (S)	24.6 [21.4; 37.1]	23.600 [20.8; 30.1]	30.4 [23.9; 49.9]	**<.001**
Fibrinogen (g/L)	2.6 [2.2; 2.9]	2.620 [2.3; 2.9]	2.4 [2.0; 2.9]	**.039**
D-dimer (μg/mL FEU)	2.6 [1.5; 5.7]	2.335 [1.3; 4.5]	3.3 [2.2; 7.4]	**<.001**

Abbreviations: WBC, white blood cell; RBC, red blood cell; ALT, alanine aminotransferase; AST, aspartate aminotransferase; LDH, lactate dehydrogenase; Globulin, serum globulin; Albumin, serum albumin; TBIL, total bilirubin; DBIL, direct bilirubin; IBIL, indirect bilirubin; HCO_3_^−^, bicarbonate; eGFR, estimated glomerular filtration rate; cTnI, cardiac troponin I; CK-MB, creatine kinase-MB; CK, creatine kinase; hsCRP, high-sensitivity C-reactive protein; PCT, procalcitonin; IL-1β, interleukin-1 beta; IL-2R, interleukin-2 receptor; IL-6, interleukin-6; IL-8, interleukin-8; IL-10, interleukin-10; TNF-α, tumor necrosis factor-alpha; APTT, activated partial thromboplastin time; PT, prothrombin time; TT, thrombin time.

Continuous variables are presented as mean ± standard deviation [21] (SD) if normally distributed, or as median [interquartile range, IQR] if non-normally distributed. Categorical variables are presented as n (%).

Comparisons were made using the independent *t*-test for normally distributed variables, the Mann–Whitney *U* test for non-normally distributed variables, and the χ^2^ test for categorical variables. *P* < .05 was in bold and considered statistically significant.

### Laboratory Markers Associated With Fatal Outcomes in Patients With SFTS

**Figure 1.**
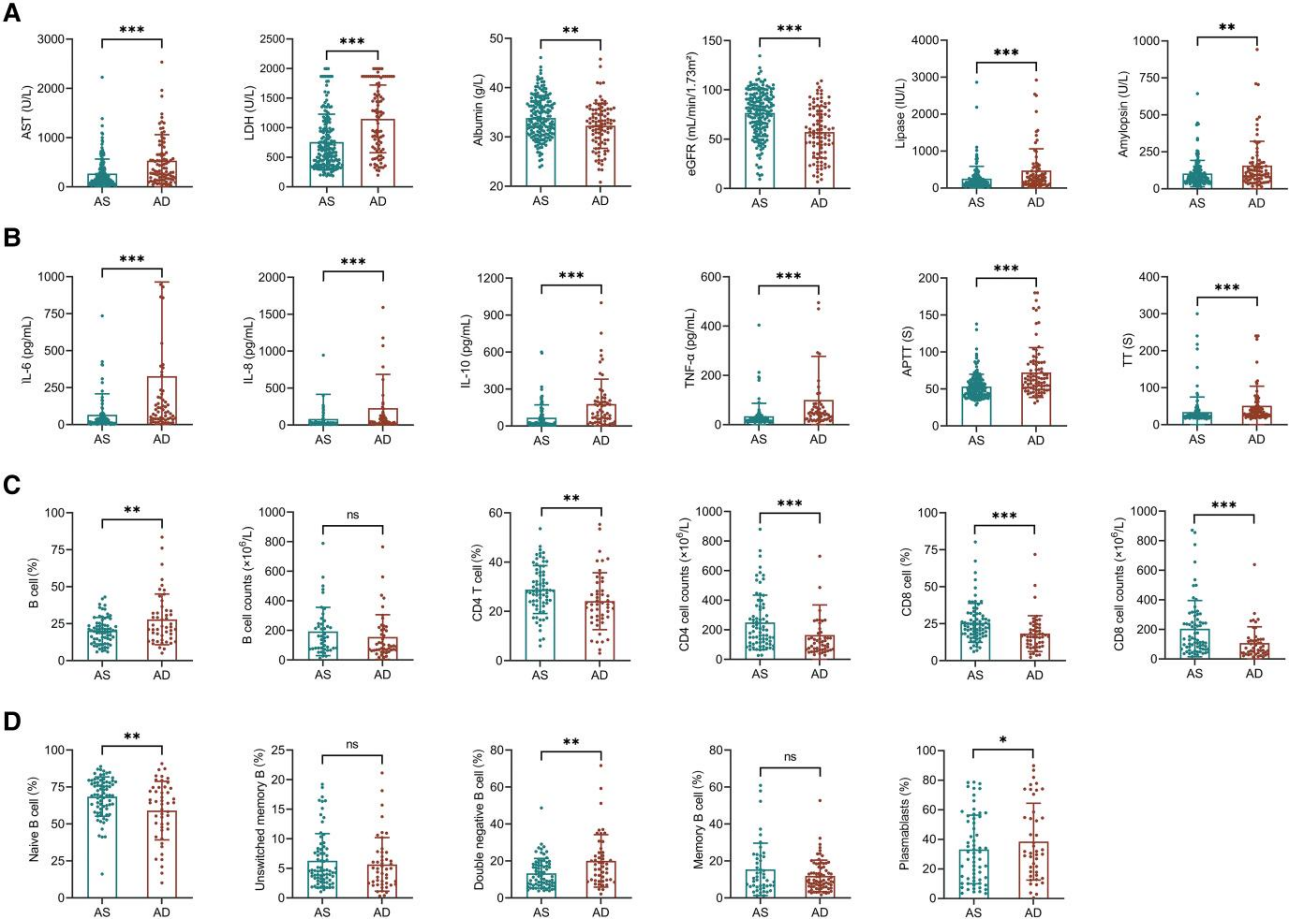
Comparison of clinical, inflammatory, and immunological indicators between survival (AS) group and deceased (AD) group of SFTS patients. *A*, Liver function and renal function markers between AS and AD groups. *B*, The comparison of inflammatory and coagulation parameters. *C*, Peripheral lymphocyte subsets between the groups. *D*, B-cell subpopulations and plasmablasts between the groups.

Laboratory analysis revealed significant differences between survival and deceased patients with SFTS, reflecting extensive systemic dysregulation in fatal cases. Deceased individuals exhibited lower platelet counts and elevated levels of liver enzymes (AST, LDH), along with indicators of renal impairment, including reduced eGFR (Figure 1*A*). Coagulation abnormalities, such as prolonged prothrombin time and APTT, were more pronounced in nonsurvivors, accompanied by elevated inflammatory markers including IL-6, IL-8, IL-10, and TNF-α (Figure 1*B*). TGF-β1 levels were significantly lower in deceased patients compared with survivors, but showed no significant difference between patients with and without neurological symptoms (Supplementary figure 1a, b). Immunologically, nonsurvivors exhibited significantly reduced absolute counts of CD3+, CD4+, and CD8+ T cells, indicating profound suppression of cellular immunity. In contrast, the total proportion of B cells was increased (Figure 1*C*). However, subset analysis revealed an abnormal B-cell distribution, characterized by a reduction in naïve B cells and significant increases in plasmablasts and double-negative (IgD-CD27-) B cells (Figure 1*D*), suggesting early and excessive B-cell activation, impaired differentiation, and potential humoral immunity.

**Figure 2.**
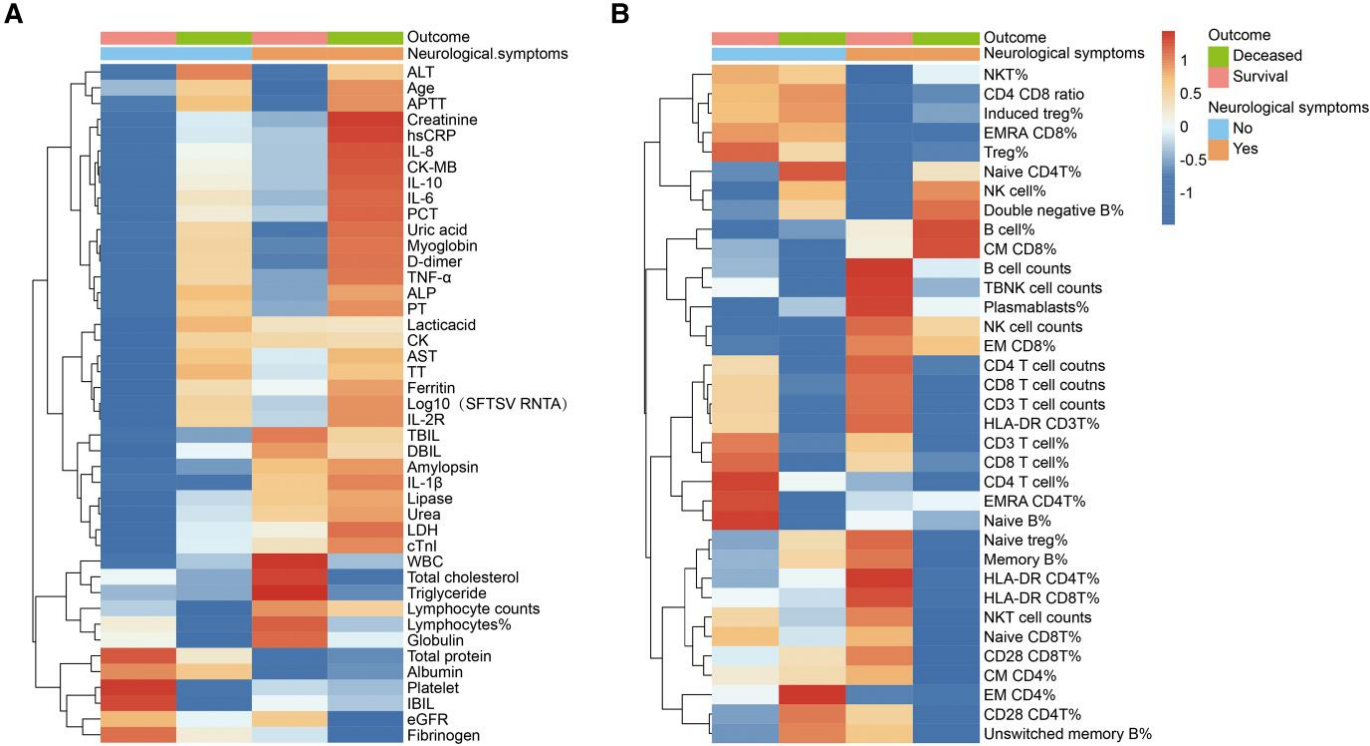
Heatmap analysis of clinical, biochemical, and immunological indicators in SFTS patients. *A*, Inflammatory cytokine levels, viral load, and coagulation parameters in SFTS patients with and without neurological symptoms and between survivors and deceased individuals. *B*, Immune cell subset distribution in SFTS patients based on survival status and neurological involvement.

Furthermore, heatmap analysis demonstrated that patients with neurological symptoms, particularly those who died, exhibited markedly higher levels of inflammatory cytokines (IL-6, IL-10, and TNF-α), increased viral loads (SFTSV RNA), and more severe coagulation disturbances (Figure 2*A* and *B*). These findings underscore the crucial roles of hyperinflammation, viral burden, immune dysfunction, and coagulation abnormalities in the pathogenesis and fatal outcomes of SFTS, especially in patients presenting with neurological involvement. Early recognition of these features may facilitate timely and targeted clinical interventions.

### Association of Neurological Symptoms and SFTSV Viral Load With Clinical Outcomes in Patients With SFTS

**Figure 3.**
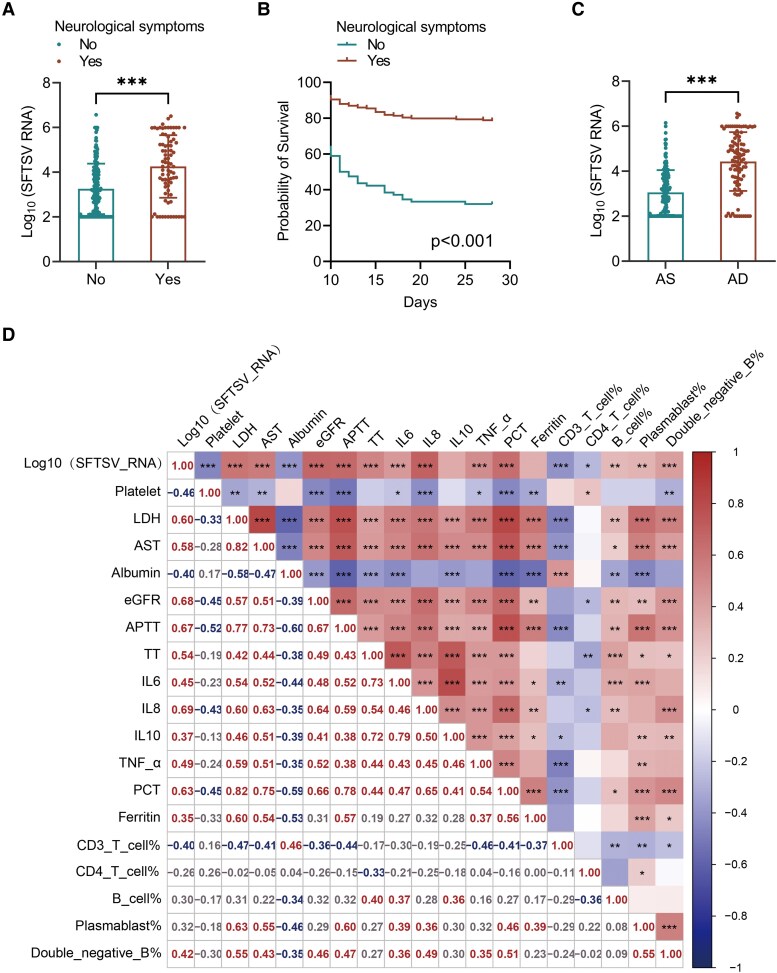
Association of neurological symptoms and SFTSV viral load with clinical outcomes in SFTS. *A*, Comparison of SFTSV viral load between patients with and without neurological symptoms. *B*, Kaplan–Meier survival curves showing significantly lower survival probability in patients with neurological symptoms. *C*, SFTSV viral load between survivors (AS group) and nonsurvivors (AD group). *D*, Correlation heatmap showing associations between viral load and key laboratory parameters, including platelet count, inflammatory cytokines, immune markers and coagulation indicators.

**Figure 4.**
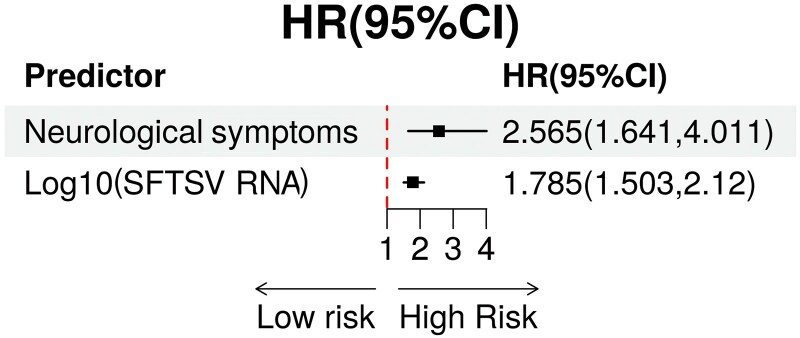
Neurological symptoms and viral load predict mortality of SFTS patients.

Patients with neurological symptoms exhibited significantly higher SFTSV viral loads compared with those without (median log_10_ SFTSV RNA: 6.8 [IQR: 5.2–8.1] vs 4.5 [IQR: 3.1–6.3], *P* < .001; Figure 3*A*). Kaplan–Meier survival analysis demonstrated a markedly lower 28-day survival probability in patients with neurological symptoms (32.05%) compared with those without (78.39%, log-rank *P* < .001; Figure 3*B*). Similarly, deceased patients (AD group) had significantly higher viral loads than survivors (AS group) (*P* < .001; Figure 3*C*). Correlation analysis revealed that elevated viral load was significantly associated with several key laboratory abnormalities, including lower platelet counts, increased levels of IL-6, IL-10, TNF-α, prolonged APTT, increased D-dimer levels, and reduced eGFR (Figure 3*D*), suggesting a close link between viral, immune activation, coagulopathy, and organ dysfunction. Multivariate Cox regression analysis further confirmed that neurological symptoms (HR = 2.565, 95% CI: 1.641–4.011, *P* < .001) and higher SFTSV viral load (HR = 1.785 per log10 increase, 95% CI: 1.503–2.120, *P* < .001) were independent predictors of 28-day mortality (Figure 4). These findings underscore the prognostic importance of both CNS involvement and vital replication in the clinical course of SFTS.

## DISCUSSION

This study demonstrated that neurological involvement plays a pivotal role in determining disease severity and clinical outcomes in patients with SFTS. Patients who developed neurological symptoms exhibited significantly higher mortality rates and more severe disease presentations, underscoring the importance of early recognition and targeted management of CNS complications. These findings are consistent with previous studies identifying neurological manifestations, particularly encephalitis, as key indicators of poor prognosis in SFTS [17, 22]. Importantly, this study builds upon existing knowledge by comprehensively integrating clinical and laboratory parameters, including immune-related biomarkers, thereby offering a more refined and detailed evaluation of prognostic factors.

One of the most important findings of this study is the observed association between elevated SFTSV RNA viral loads and neurological involvement, both of which were independently associated with increased mortality risk [11, 16]. While prior studies have reported a correlation between high viral load and disease severity [15], the present study further emphasizes the synergistic interplay between CNS complications and viral burden. This relationship suggests the potential for direct viral neuroinvasion and/or indirect CNS injury mediated by systemic immune dysregulation. The significantly elevated levels of pro-inflammatory cytokines, including IL-6, IL-10, TNF-α, and ferritin, among patients with neurological manifestations support the hypothesis of cytokine-mediated neurotoxicity, thereby extending current knowledge of cytokine storm mechanisms in severe viral infections.

The comprehensive analysis integrating laboratory and immunological parameters provides important insights into the systemic pathophysiology underlying CNS involvement and adverse outcomes in SFTS. Patients with fatal outcomes consistently exhibited significant coagulation disturbances, renal impairment, and elevated markers of organ injury. These findings are consistent with previous studies reporting multi-organ dysfunction and coagulopathy as hallmarks of severe SFTS [20, 23]. These abnormalities coincided with heightened levels of inflammatory cytokines, particularly IL-6, IL-10, TNF-α, and ferritin, which are known mediators of cytokine storm syndromes [12, 24]. Excessive cytokine release has been implicated in blood-brain barrier disruption and subsequent neuroinflammation in viral infections, suggesting that the neurological complications observed in SFTS may result not only from direct viral neurotropism, but also from immune-mediated CNS injury. Previous experimental and clinical studies have demonstrated that elevated IL-6 and TNF-α levels can increase blood-brain barrier permeability, promote leukocyte infiltration into the CNS, and exacerbate neuronal damage [25, 26]. Consistent with prior findings in fatal SFTS and severe COVID-19, we observed significantly reduced TGF-β1 levels in nonsurvivors, supporting the notion of impaired immunoregulatory control during disease progression [27, 28]. This imbalance between elevated IL-6/IL-10 and suppressed TGF-β1 may exacerbate systemic inflammation and contribute to poor clinical outcomes.

Although our study identified significant elevations of IL-6, IL-8, IL-10, and TNF-α in patients with severe outcomes, the specific cellular sources of these cytokines could not be determined within our current design. Previous studies have identified monocytes and macrophages as primary sources of IL-6, IL-10, and TNF-α during viral infections, while IL-8 is typically secreted by macrophages and endothelial cells [29–31]. Effector T cells are known to secrete pro- and anti-inflammatory cytokines, including TNF-α and IL-10, in response to viral or other pathogenic stimulation [32]. Moreover, our recent single-cell RNA sequencing study demonstrated that plasmablasts from SFTS patients can produce TNF-α and IL-6, suggesting a potential role for B cells in driving cytokine-mediated inflammation and immune dysregulation [21]. Future research utilizing single-cell transcriptomics or intracellular cytokine staining is warranted to accurately identify the cellular sources of inflammatory cytokines in SFTS and clarify their contribution to CNS injury. Furthermore, hyperferritinemia, often seen in HLH syndrome, is associated with severe systemic inflammation and adverse neurological outcomes [33].

Immunological dysregulation may play a pivotal role in the pathogenesis of severe SFTS and its associated CNS complications. Previous studies have shown that fatal viral infections are frequently accompanied by lymphopenia and T-cell exhaustion, both of which compromise antiviral immunity and contribute to systemic inflammation [9, 24, 34]. Consistent with this, our findings indicate profound suppression of cellular immunity in severe SFTS, reflected by reduced in CD4+ and CD8+ T-cell populations. Although an overall increase in B-cell proportion, the skewed distribution, characterized by reduced naïve B cells and increased plasmablasts and double-negative B cells, suggests dysregulated humoral immunity. B cells have been identified as primary targets of SFTSV infection, and transient plasmablast expansion has been associated with ineffective antibody responses, contributing to poor outcomes in severe SFTSV infection [35–37]. These immune features may facilitate viral persistence, enhance cytokine production and disrupt the blood-brain barrier, thereby increasing the risk of CNS injury. The combined impairment of T-cell surveillance and maladaptive B-cell responses may represent a central immunopathogenic mechanism in fatal SFTS.

In light of these findings, it is also important to evaluate how our results may inform and enhance current prognostic frameworks for SFTS. The prognostic value of the identified clinical and laboratory parameters is further validated by our multivariate Cox regression analysis, which identified both neurological manifestations and viral load as independent predictors of mortality. These results not only corroborate previous evidence regarding the association between high viral burden and disease severity [28], but also highlight the often-overlooked impact of CNS involvement on patient outcomes in SFTS. Most existing prognostic models for SFTS primarily incorporate general clinical indicators such as age, platelet count, or hepatic function markers [18, 38, 39]. Although these variables contribute to risk assessment, they frequently omit neurological symptoms and immune-inflammatory markers, which, as our findings suggest, are critically associated with disease progression and fatal outcomes. By integrating parameters reflective of immune dysregulation (eg, cytokine elevations, ferritin levels), organ dysfunction, and CNS complications, our study provides a more comprehensive framework for risk stratification. This integrative approach may significantly enhance the predictive accuracy of existing models, enabling earlier recognition of high-risk patients and facilitating more targeted clinical interventions. Due to the retrospective design, dynamic temporal profiles of viral load and cytokine levels were not available. As such, we could not conclusively determine whether neurological symptoms were preceded or triggered by peak viremia or immune activation. This limits causal inference and highlights the need for prospective studies with serial monitoring of viral kinetics and host immune responses.

This study has several notable limitations. First, its single-center retrospective design inherently limits generalizability and increases susceptibility to selection bias, necessitating validation through multicenter prospective cohorts. Second, the absence of CSF analyses and neuroimaging data precludes direct assessment of neurotropic viral invasion and CNS-specific pathological changes. Third, while we adjusted for key covariates, residual confounding factors (eg, pre-existing neurological conditions or genetic predispositions) may influence the observed associations. Finally, the lack of longitudinal follow-up beyond the acute phase restricts our understanding of long-term neurological sequelae and functional recovery patterns. Future studies incorporating standardized CSF virology, advanced neuroimaging modalities, and extended neurocognitive assessments would enable more robust characterization of SFTS-related neuropathogenesis.

## CONCLUSION

In conclusion, this study highlights the pivotal role of neurological manifestations and high viral load in driving disease progression and mortality in patients with SFTS. These findings underscore the urgent need for heightened clinical awareness of CNS involvement and viral burden as key indicators of poor prognosis. Considering neurological and immune profiles in risk assessment may contribute to earlier recognition of disease severity and personalized management. Further prospective studies are warranted to elucidate the underlying mechanisms and to develop effective strategies for reducing SFTS-related mortality.

## Supplementary Material

ofaf803_Supplementary_Data

## Supplementary Data

Supplementary materials are available at *Open Forum Infectious Diseases* online. Consisting of data provided by the authors to benefit the reader, the posted materials are not copyedited and are the sole responsibility of the authors, so questions or comments should be addressed to the corresponding author.
