# Supplementary material for: Neurological Manifestations and High Viral Load as Independent Predictors of Mortality in Severe Fever With Thrombocytopenia Syndrome
Source: Open Forum Infect Dis. 2025 Dec 30;13(1):ofaf803. doi: 10.1093/ofid/ofaf803 (PMC12780883; doi:10.1093/ofid/ofaf803)
Supplement: ofaf803_Supplementary_Data [file ofaf803_supplementary_data.zip › supplementary figure legend.docx]

Supplementary figure 1: Comparison of serum TGF-β1 levels in SFTS patients stratified by (a) neurological involvement and (b) survival outcome.
