# Supplementary figures and images for: Neurological Manifestations and High Viral Load as Independent Predictors of Mortality in Severe Fever With Thrombocytopenia Syndrome
Source: Open Forum Infect Dis. 2025 Dec 30;13(1):ofaf803. doi: 10.1093/ofid/ofaf803 (PMC12780883; doi:10.1093/ofid/ofaf803)

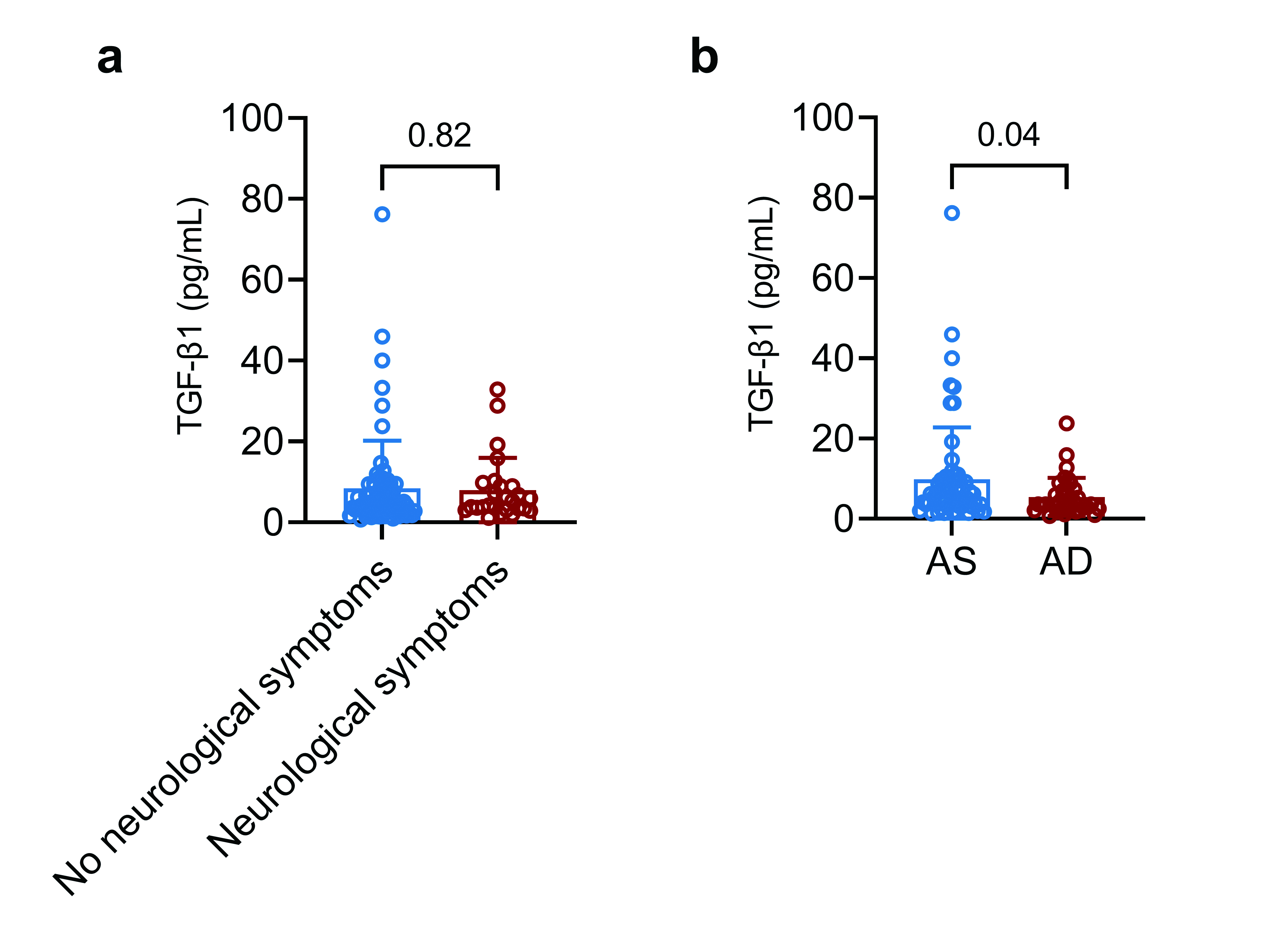

Supplement: ofaf803_Supplementary_Data [file ofaf803_supplementary_data.zip › F S1.tif]
